# Reduction of Post-Surgical Facial Edema Following Bromelain and Coumarin Intake in Traumatology: A Prospective Study with 100 Patients

**DOI:** 10.3390/jcm13040922

**Published:** 2024-02-06

**Authors:** Giuseppe Consorti, Gabriele Monarchi, Mariagrazia Paglianiti, Enrico Betti, Paolo Balercia

**Affiliations:** 1 Division of Maxillofacial Surgery, University Hospitals of Ancona, 60126 Ancona, Italy; enrico.betti@ospedaliriuniti.marche.it (E.B.); paolo.balercia@ospedaliriuniti.marche.it (P.B.); 2 Department of Medicine, Section of Maxillo-Facial Surgery, University of Siena, Viale Bracci, 53100 Siena, Italy; gabriele.monarchi@gmail.com (G.M.); m.paglianiti@student.unisi.it (M.P.)

**Keywords:** facial swelling, bromelain, post-surgical edema, pain, traumatology, facial trauma

## Abstract

**Background:** Bromelain and coumarins are recognized as safe and effective therapeutic agents, used by individuals to treat ailments such as postoperative edema, inflammation and other diseases. Bromelain has been proven to be well absorbed by the body after oral administration, and it has no major side effects even after prolonged use. The purpose of this study is to evaluate the effectiveness of bromelain and other nutraceuticals in reducing post-surgical swelling, pain and the need of anti-inflammatory drugs in maxillofacial post-traumatic surgery. **Methods:** This prospective open-label study was conducted on patients undergoing surgery for trauma of the maxillofacial area. One hundred patients were selected and divided into two groups: one group who underwent therapy with bromelain, *Aesculus hippocastanum* and *Melilotus officinalis* and a control group that was not given the drug in postoperative therapy. **Results:** Patients in the experimental group showed a reduction of edema in the first and second postoperative weeks, a faster complete reduction of facial edema and a lower reduction in maximum mouth opening and needed less anti-inflammatory therapy to control pain. **Conclusions:** These findings seem to provide evidence that Brovas^®^ may be effective in improving postoperative edema outcomes in patients undergoing surgical treatment of facial fractures.

## 1. Introduction

The field of traumatology represents an important part of maxillofacial surgery, with almost half of all surgical interventions occurring in the traumatology branch. The management of patients suffering from a fracture of single or multiple facial bones begins with the correct initial evaluation, an adequate surgical intervention for the reduction and stabilization of the fractures and a postoperative management that allows the patient to recover as soon as possible from pre-trauma morphology and functionality, minimizing the negative effects of the intervention [1].

For patients undergoing facial trauma surgery, there is a high rate of pre- and postoperative pain, worsening facial edema in the first three days, functional limitations in buccal opening, chewing, eyelid opening, etc. (depending on the region of the facial skeleton involved) [1]. 

As a result of tissue incision, skeletonization and traction forces applied to reduce fractures, facial edema is one of the constant elements in facial trauma surgery.

Worldwide, bromelain is recognized as a safe and effective therapeutic agent, used by individuals to treat ailments such as arthritis, sinusitis, bronchitis and inflammation [2].

There are many therapeutic benefits associated with bromelain, and an important indication for bromelain therapy is the reduction of postoperative and post-traumatic swelling and pain [3].

A number of researchers have also evaluated bromelain’s anti-inflammatory effects after third molar surgery, but the results have not been consistent [4]. A study by de la Barrera-Nunez et al. [5] found that bromelain reduced inflammation and improved mouth opening motion after surgery, but the results were not statistically significant. Majid and Al-Mashhadani [6], on the other hand, reported that bromelain could significantly reduce postoperative pain and swelling after third molar surgery. An objective of the present study was to provide evidence-based results regarding bromelain and coumarin’s effects on reducing postoperative symptoms and signs after facial trauma surgery. In this study, the authors hypothesized that bromelain and coumarins could significantly reduce postoperative discomforts.

In the present work, 100 patients undergoing surgical treatment for fractures of the face at the maxillofacial surgery department of Ancona Hospital (Italy) were analyzed and divided into two groups depending on whether or not they had postoperatively taken a product specifically aimed at reducing edema, pain and most of the classic disorders of the postoperative period.

In fact, the aim of this study is to evaluate the reduction of post-surgical edema with or without the intake of a product characterized by high-dose bromelain, *Melilotus officinalis* titrated at 20% in coumarin and *Aesculus hippocastanum* titrated at 20% in coumarin (Brovas^®^, AGAVE group; Bologna, Italy) in patients undergoing surgery for the reduction and stabilization of facial fractures.

## 2. Materials and Methods

The present work is a prospective open-label trial. One hundred patients undergoing a procedure for the reduction and stabilization of fractures of the facial mass were included. The patients were randomly divided into two groups (50 patients per group) through a randomized system: group A patients who did not take the product in question in the immediate postoperative period and for the agreed period and group B patients who took the product immediately after the operation and for the agreed period (1 tablet, 2 times a day for 15 days).

The inclusion criteria implemented in the present study are as follows: patients treated surgically for fracture of the facial mass, not previously operated on for the same fractures, compliant with medical instructions, of any age and of both sexes, patients with guaranteed follow-up at least 30 days post-surgery and patients who were operated on in a time window between 5–7 days after the traumatic event. However, patients who were already treated for the same fractures, non-compliant, treated in emergency or after more than 8 days, allergic to the active ingredient of bromelain and coumarin or with alterations in their state of consciousness were excluded from this study.

Both groups received the following general instructions: 15 days of light diet, brushing teeth 3 times a day and 7 days of broad-spectrum antibiotic therapy, decubitus with backrest at 30° and application of ice for 15 min alternating for the first postoperative day. This study was conducted in an open-label manner. The VAS scale was taken as a reference to assess pain and how many days of nonsteroidal anti-inflammatory therapy (NSAID) were needed to control pain during the week. The surgeons collected all measurements and clinical results before surgery, the first 24 h postoperation and seven and fifteen days after surgery.

As far as edema is concerned, in addition to a subjective evaluation of the imbibition of the soft tissues of the face which obviously presents possible operator-dependent variability, some pre-established points of the patient’s face were taken as a reference divided by facial thirds (upper, middle and lower thirds). The variations in distance between pre- and postoperative measurements were calculated in order to objectively evaluate the presence of more or less marked edema according to the increase in distance between two points. The subdivision of the face into three parts is necessary, considering the extreme heterogeneity of traumas of the facial mass. The division into three parts was carried out by taking as landmarks two parallel lines passing through the nasion, defining the upper and middle thirds, and through the base of the nostrils, defining the middle and lower thirds.

Six main points were identified: point A at the level of the lateral canthus, B at the level of the mandibular angle, C at the level of the internal canthus, D at the level of the midpoint of the chin, E at the level of the tragus and F at the level of the base of the nasal wing.

The distances measured and analyzed were AB, BC, AD, CD and BF for the lower third and EA, EC and EF for the middle third. For the upper third of the face, the pre and postoperative circumference of the patient’s head at the level of the glabella was taken as a parameter.

Additionally, the buccal opening was assessed at time 0 and after 7 and 15 days in patients with injuries to the middle and lower thirds of the face.

## 3. Statistical Analysis

Statistical analysis was performed using Stata ver. 17.0 software (StataCorp, College Station, TX, USA). The following clinical variables were analyzed in the study: age, sex, type of facial fracture, smoking habit, pain reported via VAS scale, number of anti-inflammatories taken and distances between pre-determined facial points.

Intergroup comparisons of the Wilcoxon–Mann–Whitney rank sum test using Fisher’s exact *p*-value were used.

Cohen’s D of the VAS for pain at follow-up between groups was used for power estimation. Setting alfa = 0.05, the computed power was 99%.

The Shapiro–Wilk test showed normal distribution for VAS for pain. The unpaired Student’s *t*-test was conducted for intergroup comparisons at each timepoint for VAS for pain.

The Shapiro–Wilk test showed non-normal distribution for many series of facial landmarks; thus, the Mann–Whitney rank sum test was used for comparisons.

## 4. Results

The present study includes patients who underwent surgery to reduce and stabilize facial fractures within and no later than 5–7 days from the injury and who had not previously undergone surgery for the same fracture. A total sample of 100 patients were included, divided into two groups of 50 patients each. As regards gender, 68 patients were male and 32 were female, with a ratio of almost 2:1. The mean age of both groups is 41.42 years (values between 15 and 80 years). The average age of group A is 39.8 years old while that of group B is 43.04 years old. The history of cigarette smoking was similar between the two groups with 41 non-smoking patients, 48 habitual smokers and 11 sporadic or occasional smokers. Between smokers and non-smokers, no significant differences were observed in the reduction of postoperative edema (Table 1).

As regards the face portion involved in the traumatic event, no differences were reported between the two groups. Among the 100 patients analyzed, 48 came to our attention for fractures of the lower third of the face, 38 for the middle third and 14 for the upper third. The total surgical time averaged 50.25 min (with values ranging between 15 and 220 min) and did not differ significantly between the two groups. (51.5 min for group A and 49 min for group B) (Table 2).

As regards the study of the efficacy of the medication under examination, the intake of pain-relieving/anti-inflammatory drugs (NSAIDs) in the immediate postoperative period was evaluated among the parameters. The number of intakes, the type of medication and the overall duration of treatment were evaluated. Intergroup comparisons of the Wilcoxon–Mann–Whitney rank sum test using Fisher’s exact *p*-value were used. The duration of the analgesic treatment differed significantly, with lower values in group B (median: 2, IQR: 2.3, *p* < 0.0001). The median of group A is equal to 4 days of anti-inflammatory treatment while the median of group B is equal to 2 days. The main medication used as an anti-inflammatory was ibuprofen, in 78% of cases (Table 3).

In the analysis of postoperative pain, the VAS pain scale was used, with values from 0 to 10, submitted to the patient at time 0 (pre-operative) on the first postoperative day and at 7 (T1) and 15 days (T2) after surgery.

Cohen’s D of the VAS for pain at follow-up (T2) between groups was used for power estimation. Setting alfa = 0.05, the computed power was 99%.

**Figure 1:**
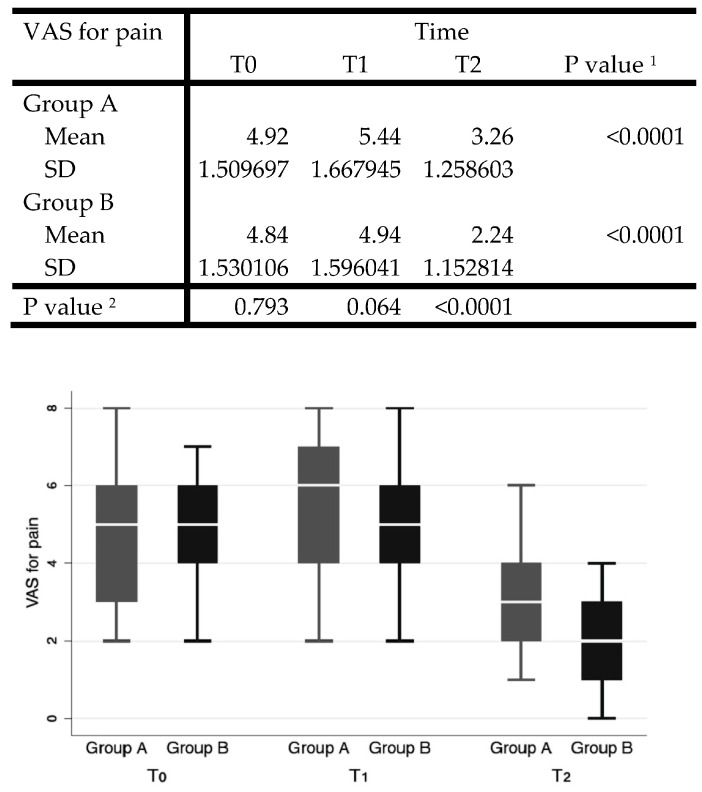
Shapiro–Wilk test showed normal distribution for VAS for pain. ^1^ Oneway ANOVA for repeated measures for single group over time. ^2^ Unpaired Student’s *t* test at each time point, verified by rank sum test.

The Shapiro–Wilk test showed normal distribution for VAS for pain, except for T0. VAS decreased significantly over time in both groups (*p* < 0.0001). The unpaired Student’s *t*-test was conducted for intergroup comparisons at each timepoint, showing significantly lower values for group B with a value of 3.26 ± 1.26 versus the value of 2.24 ± 1.15 for group A (*p* < 0.0001) (Figure 1).

**Figure 2:**
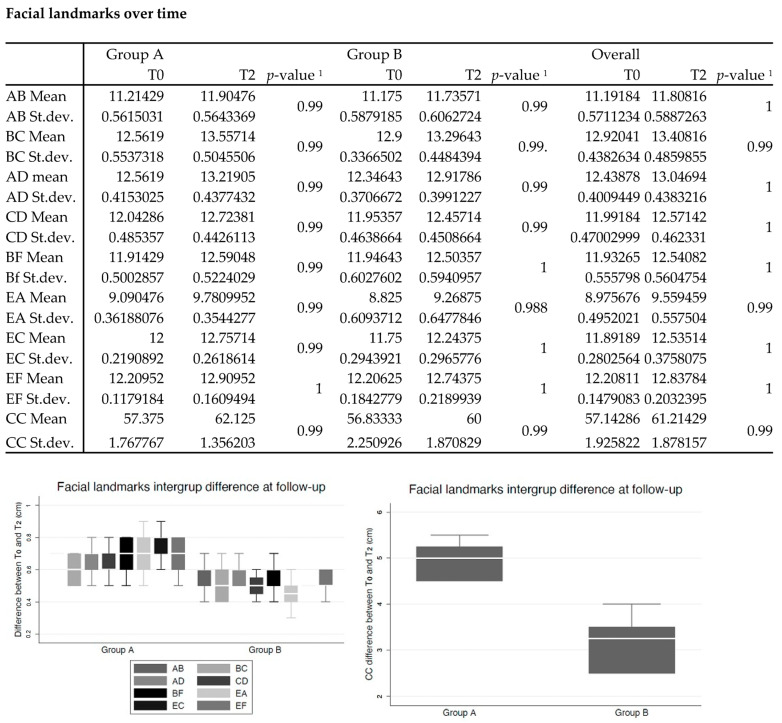
Intergroup comparison showed that T2–T0 difference was significantly lower in group B than in control for all landmarks. ^1^ Wilcoxon Mann Whitney rank sum test for intragroup over time comparison (T2 < T0) using Fisher’s exact *p* value.

As regards the evaluation of facial edema through the study of the various distances between predefined facial points, the statistical analysis revealed the following overall averages of the distances: AB difference equal to 3.181; BC difference of 18.163; AD difference of 3.405; CD difference of 3.481; BF difference of 1.687; EA difference equal to 1.463; EC difference of 2.20; EF difference of 1.327; and CC difference of 0.623 (Figure 2).

The Shapiro–Wilk test showed non-normal distribution for many series of facial landmarks; thus, the Mann–Whitney rank sum test was used for comparisons. Intergroup comparison showed that the T2–T0 difference was significantly lower in group B than in control for all landmarks (CC distance difference is plotted as a separate graph as range was on difference scale).

The mean buccal opening reported at T0 was 3.7 cm for both groups. At T1, we recorded a buccal opening in group A patients equal to 4.2 cm and in group B equal to 4.7 cm. At T2 in both groups, values of 4.9 and 5.1 cm were recorded for groups A and B, respectively, compatible with the values prior to the traumatic event.

In group B patients, no side effects of any kind related to taking the treatment or allergic reactions to the active ingredients contained in the product were found. The dosage taken by patients, i.e., one tablet every 12 h, has been shown to be effective from a therapeutic point of view and below the toxic dose of the active ingredients it contains.

## 5. Discussion

The product examined in this study contains three main active ingredients: bromelain (tit. 2400 GDU/g; 700 mg/day), *Aesculus hippocastanum* (370 mg/day) and *Melilotus officinalis* (200 mg/day).

The sulfhydryl proteolytic enzyme bromelain is found in pineapple plants and has many therapeutic applications. Numerous scientific studies have been conducted on this substance because of its low toxicity, high efficiency, high availability and relative simplicity of acquisition [7].

Despite numerous studies regarding bromelain’s biochemical characteristics, the molecular mechanisms responsible for its activity remain largely unknown.

An important indication for bromelain therapy is the reduction of postoperative and post-traumatic swelling [8,9]. Previous studies have shown a correlation of this phytotherapeutic medicine with decreases in COX2 concentration [10].

The treatment administered in this study contains bromelain in high doses, 700 mg/day, and high concentrations, tit. 2400 GDU/d; moreover, tablets have gastroprotection, allowing them to reach the intestine without being largely degraded in the stomach and absorbing in the maximum quantity possible, ensuring an effective dose for multiple systemic functions.

The coumarins extracted from horse chestnuts contribute to maintaining the normal functionality of the microcirculation and reduce edema. In particular, the coumarin derivative *Aesculus hippocastanum* has proven anti-inflammatory properties [11].

It has been shown that natural and synthetic coumarins exhibit a wide range of biological activities. Moreover, several coumarin derivatives have been shown to be anticoagulant, antioxidant, antiangiogenic, anticancer and antibacterial. The clinical use of certain derivatives of coumarins includes anticoagulants (warfarin, antivitamin K compounds) and treatments for psoriasis (methoxsalene). Coumarins have been extensively investigated for their potential application in the development of anti-inflammatory drugs. Various coumarin derivatives have also been shown to inhibit inflammation via different mechanisms [12].

*Melilotus officinalis*, titrated at 20% in coumarins, is a plant known for promoting the normal functionality of microcirculation, venous circulation and drainage of body fluids.

These three active ingredients, therefore, cooperate in reducing post-surgical edema and in relieving major post-surgical treatment ailments.

These compounds have become of importance in recent years due to their various biological activities. Previous biological activity studies performed on coumarin derivatives revealed that these compounds have anti-edema, antitumor, photochemotherapy, anti-HIV, antibacterial, antifungal, anti-inflammatory, anticoagulant, triglyceride-lowering and central nervous system-stimulating effects [12,13].

Studies comparing bromelain to diclofenac, or other NSAIDs, in controlling muscular pain found no significant differences [7]. Another study pointed out a notable effect of bromelain on reducing knee algesia in patients with arthritis [14]. These results are justified by the fact that bromelain reduces pain-inducing mediators, such as bradykinin [15] and factors (edema, debris and immune complexes).

On the other hand, it should be noted in the same studies that, despite a trend in pain reduction on the first and third postoperative days, the results were not significant in the statistical analysis.

Bromelain showed, through the results of this study, that it has the capacity to minimize postoperative trismus. Other studies have not shown the same results in terms of medication efficacy [16,17].

Possibly, different aetiologies for trismus and edema explain different results.

In our analysis, all the limitations recognized were attributed to edema and trismus as well as to pain [4].

In the case of lower third molar surgery, bromelain appears to reduce pain, swelling and trismus.

Despite the limited number and quality of available studies and their high heterogeneity, we cannot conclude that bromelain is an effective treatment [18].

In contrast, the isolated results of the analyzed studies suggest that this might be a promising drug. As a result, the authors propose that more randomized clinical trials with methodological rigor and standardization be conducted to answer the question raised by this study.

An intention-to-treat analysis was carried out, including all patients independently from data at follow-up. A total sample of 100 patients were included in the study and allocated into two groups (group A—control group, and group B). Groups were parallel and assessed longitudinally. Intergroup comparison at baseline was carried out to check for differences in age, smoking habit, surgical time and involvement of face portion.

The subdivision of the face into three portions (upper, middle and lower face) made it possible to study fractures involving different facial districts in a uniform and standardized manner, while maintaining the ability to make objective any variation in facial edema in the same district. Furthermore, the location, the extent of the fractures and the age of the patients are comparable between the two groups analyzed.

The sample had a power of 99%, based on the post hoc Cohen’s D for primary endpoint (decrement of VAS for pain). The continuous series were checked for normal distribution by means of the Shapiro–Wilk test. Where non-normal distribution was found, a non-parametric Wilcoxon–Mann–Whitney rank sum test was implemented. Otherwise, an unpaired Student’s *t*-test was carried out. For multiple timepoints comparison (i.e., VAS for pain at each timepoint), one-way ANOVA was conducted. Categorical variables were compared between groups using a chi-squared test.

An over-time comparison (T0 vs. T2) was carried out with an alternative hypothesis that facial landmark distance decrease over time, though this was not significant for all comparisons. However, it is worth underlining that pre-operative swelling should be lower than post-surgical swelling; thus, at T2, higher values of face distance differences were measured. In consideration of this, the real underlying hypothesis should be that at T2 swelling increases less in groups using the treatment than in control groups.

In fact, an intergroup comparison showed that the T2–T0 difference was significantly lower in group B than in the control, for all landmarks.

Compared with the control group, patients who took bromelain from the immediate postoperative period reported greater overall satisfaction. The simple act of being discharged with a drug that reduces postoperative edema enhanced the quality of life of the patients. Patients examined who took bromelain and coumarins reduced their use of NSAIDs due to its anti-edema, anti-inflammatory and pain-relieving properties.

In the field of trauma surgery of the facial region, post-surgical edema is a common complaint among patients. The typical clinical picture is completed with diffuse facial pain and functional limitation. Patients mainly present difficulties in eyelid opening, buccal opening, mandibular protrusion and lateral movements, swallowing and masticatory difficulties.

Most of these symptoms resolve within 2 weeks after surgery, with the exception of facial swelling, which may take up to 4 weeks.

To achieve complete resolution of symptoms, maxillofacial surgeons should accurately quantify the extent and duration of postoperative edema [1].

In post-traumatic surgery of the facial area, there is an extremely heterogeneous range of interventions based on the severity of the injury, the facial area involved and other factors [19,20].

In maxillofacial trauma, facial edema is a constant component of the immediate postoperative period.

Therefore, maxillofacial surgeons should establish an appropriate pharmacological treatment and preventive physical measures immediately.

Since the product in this study contains an excellent profile of active ingredients, as well as considering bromelain’s very low toxicity (no evidence of toxicity at dosages greater than 3000 GDU/gr), the authors believe the product examined in this study is an excellent option for managing post-surgical edema and pain.

The most commonly reported side effects in the literature were stomach upset and diarrhea, increased heart rate, hives and throat tightness even if for very high doses and, in any case, not frequently [21]. In the present study, no side effects were reported in short-term and distant controls.

The limitation of the study lies in the fact that it was not possible to conduct it in a double-blind manner, despite the fact that we still obtained relevant data based on the objective evaluation of the edema; another limitation may be that some elderly patients do not consistently take their medication. In our study, we did not observe this problem as we ensured that patients regularly took the therapy; however, this problem could arise in a larger number of subjects analyzed.

## 6. Conclusions

Worldwide, bromelain is recognized as a safe and effective therapeutic agent, used by individuals to treat ailments such as arthritis, sinusitis, bronchitis and inflammation.

Bromelain has been proven to be well absorbed by the body after oral administration, and it has no major side effects even after prolonged use. The results obtained from this study showed a significant reduction of post-surgical edema and pain in group B compared to the control group for trauma surgery. Furthermore, the same group of patients analyzed took less oral painkillers than the control group.

Future prospects are represented by the increase in the population studied over the years and the extension of the fields of application of the intake of bromelain and coumarins in the field of orthognathic and oncological surgery.

## Figures and Tables

**Table 1:** Distribution of age, smoking habits and average duration of surgery for the two groups.

**Age**						
	**Non Missing**	**Mean**	**Standard Deviation**	**Standard Error of the Mean**	**Minimum Value**	**Maximum Value**
Age (overall)	100	41.42	15.5213	1.552	15	80
Age Group A	50	39.8	15.6622	2.2149	15	76
Age Group B	50	43.04	15.3649	2.1729	17	80
	**T**	**Mean Group A**	**SD Group A**	**Mean Group B**	**SD Group B**	***p*-Value ^1^**
Age	−1.044199	39.8	15.66225	43.04	15.3649	0.2989623
**Smoking Habit**						
**Smoke**	**Group**				***p-*Value ^1^**	
	**A**		**B**	**Total**		
0	23		18	41	0.596	
1	22		26	48		
2	5		6	11		
**Total**	50		50	100		

^1^ “Unpaired Student’s *t* test, two tails, non-significant Smoking habit Group” for the table “Age” and “Pearson’s Chi2 test, non-significant” for the table “smoking habits”.

**Table 2:** Distribution of the portions of the face affected by trauma and average duration of surgical operations in the two groups.

**Face Portion Involvement**							
**Face Third**		**Group**				***p-*Value ^1^**	
		**A**		**B**	**Total**		
Inferior		21		27	48	0.483	
Middle		21		17	38		
Superior		8		6	14		
**Total**		50		50	100		
**Surgical time**							
		**Non Missing**	**Mean**	**Standard Deviation**	**Standard Error of the Mean**	**Minimum Value**	**Maximum Value**
Surg time (overall)		100	50.25	21.63	2.163	15	220
Surg time Group A		50	51.5	27.4094	3.876	15	220
Surg time Group B		50	49	13.8136	1.953	30	80
	**T**	**Mean Group A**		**SD Group A**	**Mean Group B**	**SD Group B**	***p-*Value ^1^**
Surg time	0.5759	51.5		27.4094	49	13.8136	0.566

^1^ “Pearson’s Chi2 test, non-significant” for the table “Face portions involved” and “Unpaired Student’s T test, two tails, non-significant Smoking habit Group” for the table “Surgical Time”.

**Table 3:** Comparison of Wilcoxon–Mann–Whitney rank sum tests among groups in the treatment of postoperative pain with NSAIDs.

**Postoperative Treatment of Pain with NSAIDS’s**						
	**Non Missing**	**Median**	**25%**	**75%**	**Minimum Value**	**Maximum Value**
Analg time (overall)	100	3	2	4	1	7
Analg time Group A	50	4	3	5	2	6
Analg time Group B	50	2	2	3	1	7
	**Z**	**Observed Rank Sum**		**Expected Rank Sum**		***p*-Value ^1^**
Analg time	6.506	5050		5050		<0.0001

^1^ Wilcoxon Mann Whitney rank sum test intergroup comparison using Fisher’s Exact *p* value, highly statistically significant.

## Data Availability

Data are contained within the article.

## References

[1] Sikora M., Chlubek M., Grochans E., Jurczak A., Safranow K., Chlubek D. (2019). Analysis of Factors Affecting Quality of Life in Patients Treated for Maxillofacial Fractures. Int. J. Environ. Res. Public Health.

[2] Leelakanok N., Petchsomrit A., Janurai T., Saechan C., Sunsandee N. (2023). Efficacy and safety of bromelain: A systematic review and meta-analysis. Nutr. Health.

[3] Pezzani R., Jiménez-Garcia M., Capó X., Gürer E.S., Sharopov F., Rachel T.Y.L., Woutouoba D.N., Rescigno A., Peddio S., Zucca P. (2023). Anticancer properties of bromelain: State-of-the-art and recent trends. Front. Oncol..

[4] Ghensi P., Cucchi A., Creminelli L., Tomasi C., Zavan B., Maiorana C. (2017). Effect of oral administration of bromelain on postoperative discomfort after third molar surgery. J. Craniofacial Surg..

[5] de la Barrera-Núñez M.C., Yáñez-Vico R.M., Batista-Cruzado A., Heurtebise-Saavedra J.M., Castillo-de Oyagüe R., Torres-Lagares D. (2014). Prospective double-blind clinical trial evaluating the effectiveness of bromelain in the third molar extraction postoperative period. Med. Oral Patol. Oral Cir. Bucal.

[6] Majid O.W., Al-Mashhadani B.A. (2014). Perioperative bromelain reduces pain and swelling and improves quality of life measures after mandibular third molar surgery: A randomized, double-blind, placebo-controlled clinical trial. J. Oral. Maxillofac. Surg..

[7] Chakraborty A.J., Mitra S., Tallei T.E., Tareq A.M., Nainu F., Cicia D., Dhama K., Emran T.B., Simal-Gandara J., Capasso R. (2021). Bromelain a Potential Bioactive Compound: A Comprehensive Overview from a Pharmacological Perspective. Life.

[8] Bormann K.H., Weber K., Kloppenburg H., Staude P., Koch A., Meiser P., Gellrich N.C. (2016). Perioperative Bromelain Therapy after Wisdom Teeth Extraction—A Randomized, Placebo-Controlled, Double-Blinded, Three-Armed, Cross-Over Dose-Finding Study. Phytother. Res..

[9] Singh T., More V., Fatima U., Karpe T., Aleem M., Prameela J. (2016). Effect of proteolytic enzyme bromelain on pain and swelling after removal of third molars. J. Int. Soc. Prev. Community Dent..

[10] Leal L.E., Moreira E.S., Correia B.L., Bueno P.S.A., Comar J.F., de Sá-Nakanishi A.B., Cuman R.K.N., Bracht A., Bersani-Amado C.A., Bracht L. (2023). Comparative study of the antioxidant and anti-inflammatory effects of the natural coumarins 1,2-benzopyrone, umbelliferone and esculetin: In Silico, in vitro and in vivo analyses. Naunyn-Schmiedeberg’s Arch. Pharmacol..

[11] Jančič U., Gorgieva S. (2021). Bromelain and Nisin: The Natural Antimicrobials with High Potential in Biomedicine. Pharmaceutics.

[12] Hassanein E.H.M., Sayed A.M., Hussein O.E., Mahmoud A.M. (2020). Coumarins as Modulators of the Keap1/Nrf2/ARE Signaling Pathway. Oxid. Med. Cell. Longev..

[13] Fylaktakidou K.C., Hadjipavlou-Litina D.J., Litinas K.E., Nicolaides D.N. (2004). Natural and synthetic coumarin derivatives with an-ti-inflammatory antioxidant activities. Curr. Pharm. Des..

[14] Pereira I.C., Sátiro E.E., de Oliveira Torres L.R., da Silva F.C.C., e Sousa J.M.D.C., Torres–Leal F.L. (2023). Bromelain supplementation and inflammatory markers: A systematic review of clinical trials. Clin. Nutr. ESPEN.

[15] Saptarini N.M., Rahayu D., Herawati I. (2019). Antioxidant activity of crude bromelain of pineapple (*Ananas comosus* (L.) Merr) crown from Subang district, Indonesia. J. Pharm. Bioallied Sci..

[16] Gupta A.A., Kambala R., Bhola N., Jadhav A. (2022). Comparative efficacy of bromelain and aceclofenac in limiting post-operative in-flammatory sequelae in surgical removal of lower impacted third molar: A randomized controlled, triple blind clinical trial. J. Dent. Anesth. Pain Med..

[17] Faramarzi M., Sadighi M., Shirmohamadi A., Kazemi R., Zohdi M. (2023). Effectiveness of Bromelain in the control of postoperative pain after periodontal surgery: A crossover randomized clinical trial. J. Adv. Periodontol. Implant Dent..

[18] Saptarini N., Mustarichie R., Rahayu D. (2023). Isolation, Characterization, and Evaluation of Protease Activity of Crude Bromelain of Pineapple Peel, Core, and Crown from Subang District, Indonesia. J. Pharm. Bioallied Sci..

[19] Consorti G., Betti E., Catarzi L. (2022). Customized and Navigated Primary Orbital Fracture Reconstruction: Computerized Operation Neuronavigated Surgery Orbital Recent Trauma (CONSORT) Protocol. J. Craniofacial Surg..

[20] Consorti G., Betti E., Catarzi L. (2023). Orbital Fractures: A New CT-Based Protocol to Guide the Surgical Approach and Reconstruction Material Decision-Making. J. Craniofacial Surg..

[21] Kumar V., Mangla B., Javed S., Ahsan W., Kumar P., Garg V., Dureja H. (2023). Bromelain: A review of its mechanisms, pharmacological effects and potential applications. Food Funct..

